# Inequalities in suicide mortality rates and the economic recession in the municipalities of Catalonia, Spain

**DOI:** 10.1186/s12939-015-0192-9

**Published:** 2015-09-08

**Authors:** Carme Saurina, Manel Marzo, Marc Saez

**Affiliations:** Research Group on Statistics, Econometrics and Health (GRECS), University of Girona, Girona, Spain; CIBER of Epidemiology and Public Health (CIBERESP), Madrid, Spain

**Keywords:** Suicides rates, Economic recession, Unemployment rate, Hierarchical mixed models

## Abstract

**Introduction:**

While previous research already exists on the impact of the current economic crisis and whether it leads to an increase in mortality by suicide, our objective in this paper is to determine if the increase in the suicide rate in Catalonia, Spain from 2010 onwards has been statistically significant and whether it is associated with rising unemployment.

**Methods:**

We used hierarchical mixed models, separately considering the crude death rate of suicides for municipalities with more than and less than 10,000 inhabitants as dependent variables both unstratified and stratified according to gender and/or age group.

**Results:**

In municipalities with 10,000 or more inhabitants there was an increase in the relative risk of suicide from 2009 onwards. This increase was only statistically significant for working-aged women (16–64 years). In municipalities with less than 10,000 inhabitants the relative risk showed a decreasing trend even after 2009. In no case did we find the unemployment rate to be associated (statistically significant) with the suicide rate.

**Conclusions:**

The increase in the suicide rate from 2010 in Catalonia was not statistically significant as a whole, with the exception of working-aged women (16–64 years) living in municipalities with 10,000 or more inhabitants. We have not found this increase to be associated with rising unemployment in any of the cases. Future research into the effects of economic recessions on suicide mortality should take into account inequalities by age, sex and size of municipalities.

## Introduction

Considerable research has been carried out on the impact the current economic crisis has had on health [1–12]. It is well known that economic recessions have a negative impact on mental health disorders [13–17]. Moreover, this impact could be greater among the unemployed [12, 16].

Some of the studies analysing previous economic crises have found evidence of increased numbers of suicides [18–21] and some show a clear association between the increase in unemployment and the increase in the number of suicides [18, 19, 22].

However, is there a clear causal relationship between the effects of the economic crisis and the number of suicides? Is the effect the same in all countries, and in all regions of a country [23, 24]? Exploring the impact of the current economic crisis in 54 countries, Chang *et al.* [23] showed that suicide rates are increasing both in Europe and America, and that they are higher among males and in countries with high levels of unemployment. In particular a 13.3 % increase in the number of suicides in men was demonstrated in the European Union [23]. Spain^1^ in 2009 experienced a 7.2 % increase in the suicide rate for males, but a slight decrease in females [25]. Karanikolos *et al*. [8] indicated that from 2007 suicides increased in countries such as Greece, Portugal and Spain. However, Ayuso-Mateos *et al.* [26], in response to Karanikolos *et al*. [8], pointed out that there was not an increase in Spain and Portugal, but rather a slight decrease in the number of suicides and that it was not possible to link increases in unemployment with the number of suicides.

Although previous research addresses the relationship between the increase in the number of suicides and the economic crisis, the evidence of the effects of the economic crisis on the number of suicides is still scarce. Exploring PubMed and Embase in April 2014, using the word ‘suicide’, combined with ‘economic recession’, ‘economic crisis’, ‘economic downturn’ and ‘financial crisis’ and limiting the search to articles published in the last 5 years, provided a total of 131 references, 9 of which corresponded to 2014. Most correspond to revisions or proposals for future research. Only 29 of the articles provide quantitative evidence of the relationship between periods of economic crisis and suicide, and only 16 of those made any statistical inferences [6, 7, 9, 11, 12, 23–25, 27–34].

**Fig. 1:**
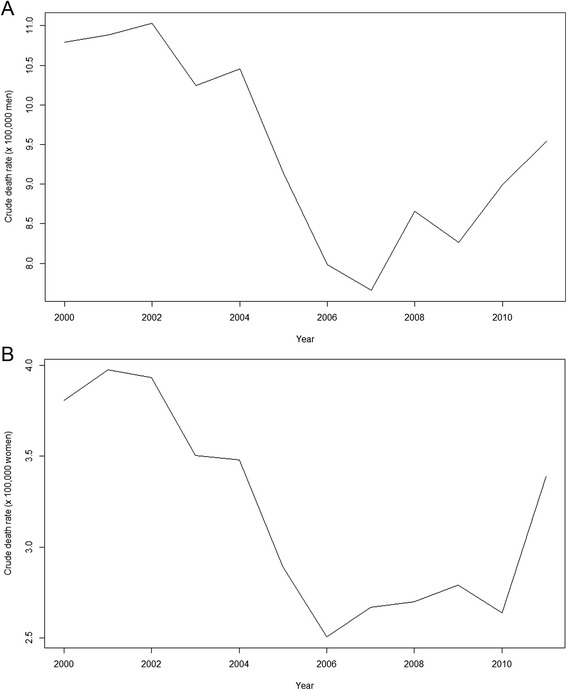
Temporal evolution of crude rates of suicide (x 100.000 people). Catalonia, 2000–2011. **a** Men. **b** Women

The suicide (crude) rates in Catalonia follow a somewhat different temporal behaviour than that of the economic recession (Fig. 1). Crude rates for men fell from 2002 to 2007 (with a spike in 2004) and then started to rise (with a temporary decrease in 2009). For women, the peak in 2004 was less abrupt and the increase from 2010 was much sharper. Note also, that in the case of women the increase in suicide rates began in 2006, a year earlier than for men. At any rate, the changing trend in suicide rates for both men and women occurs well before the recession (two years beforehand for men and three for women).

In summary, while economic recessions clearly do have a negative impact on mental health, the evidence of the effects of the economic crisis on the number of suicides is still limited and consequently rising unemployment cannot be linked definitively to the number of suicides. Our objective in this paper is twofold. First, to establish if the increase in the suicide rate, especially since 2010, is statistically significant and, second, to determine whether this increase has been associated with rising unemployment due to the economic crisis by analysing possible gender, age and size of municipalities differences.

## Methods

### Setting

The annual number of deaths in Catalonia, Spain, from 2000 to 2011 (for adults aged 16 years and over) by suicide and intentional self-harm (International Classification of Diseases (ICD-9 codes: E950-E959; ICD-10: X60-X84)) was provided by the Mortality Register of Catalonia.

#### Studied municipalities

For confidentiality reasons, data was only available for municipalities with 10,000 inhabitants or more (92 municipalities from a total of 946 municipalities in Catalonia). However, aggregated data was also available at *comarca*^2^ levels (a total of 44 *comarcas* in all Catalonia). In 2011, these 93 municipalities with 10,000 inhabitants or more comprised 77.33 % of the population of Catalonia (i.e. 5,811,938 of 7,501,853 inhabitants).

Using the information from the *comarcas* and from the municipalities of 10,000 inhabitants or more, we calculated for each *comarca* the number of suicides (total, by gender and by age group) in municipalities with less than 10,000 inhabitants. For instance, in the *comarca* of ‘Alt Camp’, there is only one municipality (Valls) with more than 10,000 inhabitants. We took the number of suicides among men for a given year in the *comarca* of ‘Alt Camp’ and subtracted the number of suicides among men in Valls^3^ for the same year. We repeated this process for each year, sex and *comarca*. Note that, in this case we considered the number of suicides in all the municipalities, with less than 10,000 inhabitants, grouped together, unlike the municipalities with more than 10,000 inhabitants.

Population data as a total, by gender and according to age group for the 946 municipalities and the 44 *comarcas* from 2000 to 2011 was provided by the Statistical Institute of Catalonia (IDESCAT). IDESCAT also provided (for municipalities of 10,000 inhabitants or more and for the *comarcas*) the number of unemployed persons (registered in employment offices), the total number (population aged 16 to 65) and by gender, although in this case from 2005 to 2011. Again, we calculated these variables in each *comarca* for the other municipalities with less than 10,000 inhabitants (as mentioned in the previous paragraph).

Finally, for the municipalities of 10,000 inhabitants or more and for the remaining municipalities with less than 10,000 inhabitants, in each *comarca* we calculated both the crude death rates from suicide as well as the unemployment rates. Note that we used the population aged 16 to 65 as the denominator for unemployment rates instead of the active population, as the latter figure was not known.

The data provided included the total number of suicides, the number of suicides by gender (3902 men and 1343 women) and according to age group (16 to 64 years and 65 years or older).

### Statistical analysis

We had, in fact, a mixed longitudinal design. Besides having two dimensions (time and municipality), this design allowed for the following points to be taken explicitly into account: i) we had units (municipalities in this case) that did not behave in the same way over time, ii) the effect of the explanatory variables on the response variable may not be the same for the different units, and iii) longitudinal observations within the same higher-level unit (municipality in this case) are not independent of one another [24]. Furthermore, we wanted to explicitly allow for the estimation of municipality time trends and the effects of the economic crisis on suicides at the municipality level. For these reasons, we used hierarchical mixed models to assess the variation in suicide rates attributable to the economic crisis as well as the association between suicide rates and unemployment (further details can be found in Saurina *et al.* [24]). In both cases, we used the (crude death) rate of suicides as the dependent variable for municipalities with more than and less than 10,000 inhabitants separately, unstratified and stratified by gender (men and women) and according to age group (16–64 and 65 and older).

Following the example of Saurina *et al.* [24] in the models we included as explanatory variables, a time trend and a dummy variable for the crisis years 2009–2011. The dummy variable was designed to capture a break from past time trends. When assessing the association between unemployment and suicide, the unemployment rate was included instead of the dummy. In this case, a time trend was also included to monitor any spurious relationship, as suicides and unemployment could evolve over time in the same way.

Both the intercept and the coefficients associated with all of the explanatory variables were considered random effects. In other words, all coefficients were allowed to vary in the higher-level unit that was considered, i.e. municipalities (in the models of municipalities with more than 10,000 inhabitants) and *comarca* (in the models of municipalities with less than 10,000 inhabitants - *areas* hereinafter). In the case of the time trend, we assumed that the random effects vary by areas and year. Thus, we used a non-parametric approach to area trends, which we assumed to evolve non-linearly.

Heteroskedasticity, that is the consequence of heterogeneity between areas, was controlled through the random intercept (at the areas level), and autocorrelation, that is the serial dependence of the longitudinal observations within the same area, was controlled in all the models through an autoregressive model of order 1.

In our case, the data contains numerous zero counts. In 77.2 % of the municipalities of over 10,000 inhabitants and 85.2 % of less than 10,000 inhabitants there was no suicide between 2000 and 2011. Typically, a Poisson model is assumed for modelling the distribution of the count observation or, at least, approximating its distribution. However, when there is an excess of zero counts, as in our case, the dispersion of the Poisson model underestimates the observed dispersion. Mixed-distribution models, such as the zero-inflated Poisson (ZIP), are often used in such cases. In particular, the zero-inflated Poisson distribution (ZIP) regression might be used to model count data for which the proportion of zero counts is greater than expected on the basis of the mean of the non-zero counts [35, 36]. In this paper we used a Type 0 ZIP. Type 0 is a mixture of a truncated Poisson (the positive observations) and a point mass at 0. This means, for instance, that Type 0 can have a lower probability at 0 than a pure Poisson.

The inferences were performed using a Bayesian approach, with the Integrated Nested Laplace Approximation (INLA) [37]. All analyses were conducted using the free software R (version 3.0.3), available through the INLA library.

## Results

**Fig. 2:**
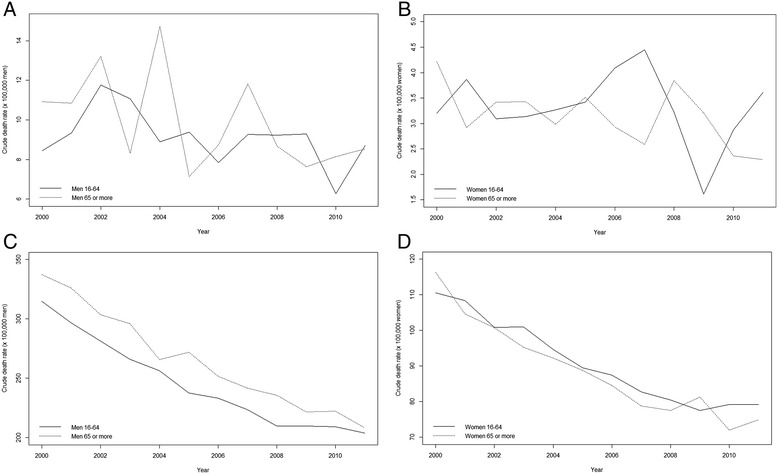
Temporal evolution of crude rates of suicide (x100,000 people). Catalonia, 2000–2011. **a** Men from municipalities whith more than 10,000 inhabitants. **c** Men from municipalities whith less than 10,000 inhabitants. **b** Women from municipalities whith more than 10,000 inhabitants. **d** Men from municipalities whith less than 10,000 inhabitants. Continuous lines for persons aged 16 to 64 years and dotted lines for people 65 and older

In municipalities with more than 10,000 inhabitants, as shown in Fig. 2a, the peak in 2004 of male suicide rates corresponded to the behaviour of the rates among men 65 years or older. This age group presented a drop from 2007 to 2009 that, probably, led to the interruption in 2009 of the increase in male suicide rates since 2007 (Fig. 1a). Furthermore, the increase from 2010 was greater in the case of men aged 16 to 64 years. An opposite temporal behaviour was observed in women between age groups 16–64 years and 65 and over (Fig. 2b). Thus, suicide rates for women aged 16 to 64 years increased from 2005 to peak in 2007 before falling from 2007 to 2009 and then once again increasing from 2009, albeit slightly less sharply from 2010 onwards. However, suicide rates among women aged 65 or older actually declined from 2005 until 2007, but then increased from 2007 to 2008 only to decline once again, although from 2010 onwards this decrease was smaller.

For municipalities with less than 10,000 inhabitants, although the rates were much higher than for municipalities with more than 10,000 inhabitants, their temporal behaviour was totally different (Fig. 2c and 2d) with a decrease from the beginning of the study period. Notice how, in this case, while the rates for men aged 65 years or more were always higher than those of men 16 to 64 years, the rates for women aged 65 or more were somewhat lower than those of women 16 to 64 years (albeit with some exceptions such as the peak of 2009).

The results of the estimation of the first model investigating the effect of the crisis on the excess number of suicides are shown in Table 1. There was only a significant increase in the suicide rates for 2011 in municipalities with more than 10,000 inhabitants among women of all ages and, particularly, women aged 16 to 64. Note, however, that it was only significant at 90 %. For other cases, although an increase in the suicide rates was observed in some cases, this was not statistically significant. For municipalities with less than 10,000 inhabitants no increase in the suicide rates was observed from 2009 onwards.

**Table 1:** Estimation results from the models assessing the effects of the economic crisis on suicide death rates. Relative risks (95 % credibility interval)

	Municipalities with 10,000 inhabitants or more		
	2009	2010	2011
All	0.866 (0.790, 0.944)	0.958 (0.879, 1.044)	1.038 (0.943, 1.144)
Men	0.857 (0.770, 0.946)	0.973 (0.883, 1.077)	1.046 (0.935, 1.174)
16-64 years	***0.881 (0.783, 0.978)***	0.972 (0.875, 1.084)	1.036 (0.914, 1.183)
≥ 65 years	***0.820 (0.671, 0.975)***	0.941 (0.787 .,1.149)	1.007 (0.816 . 1.268)
Women	***0.824 (0.677, 0.975)***	0.987 (0.832 .,1.177)	**1.157 (0.940,** **1.435)**
16-64 years	**0.833 (0.649,** **1.030)**	1.049 (0.844, 1.322)	**1.261 (0.958,** **1.676)**
≥ 65 years	0.987 (0.895, 1.020)	0.989 (0.902, 1.028)	0.991 (0.905, 1.039)
	Municipalities with less than 10,000 inhabitants		
	2009	2010	2011
All	1.000 (0.984 . 1.017)	0.996 (0.977 . 1.013)	0.992 (0.968 . 1.012)
Men	0.998 (0.980 . 1.014)	0.994 (0.973 . 1.011)	0.991 (0.965 . 1.011)
16-64 years	0.995 (0.974 . 1.012)	0.991 (0.966 . 1.010)	0.989 (0.959 . 1.011)
≥ 65 years	0.993 (0.964 . 1.013)	0.991 (0.957 . 1.013)	0.989 (0.949 . 1.015)
Women	0.995 (0.971 . 1.013)	0.992 (0.963 . 1.013)	0.990 (0.955 . 1.013)
16-64 years	0.993 (0.964 . 1.013)	0.991 (0.957 . 1.013)	0.989 (0.949 . 1.015)
≥ 65	0.993 (0.964 . 1.013)	0.991 (0.957 . 1.013)	0.989 (0.949 . 1.015)

In bold, the 90 % credibility interval did not contain the unit. In bold and *shaded*, the 95 % credibility interval did not contain the unit

**Fig. 3:**
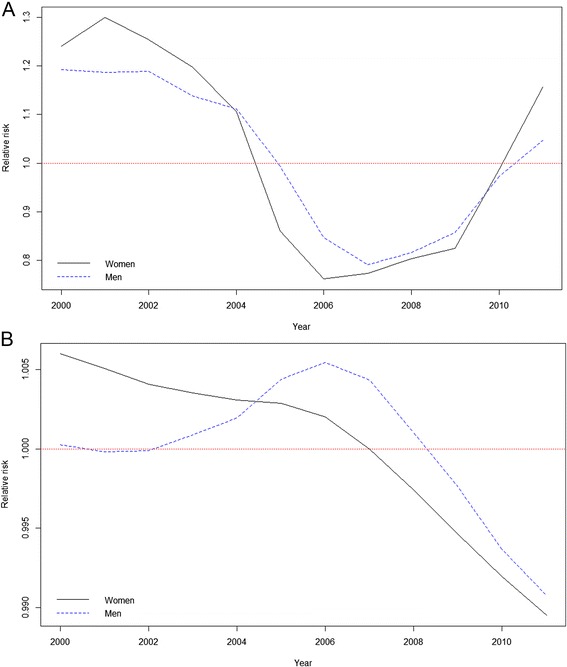
Relative risk of suicide rates. **a** Municipalities whith 10,000 inhabitants or more. **b** Municipalities whith less than 10,000 inhabitants. Dotted lines for men and continuous lines for women

**Fig. 4:**
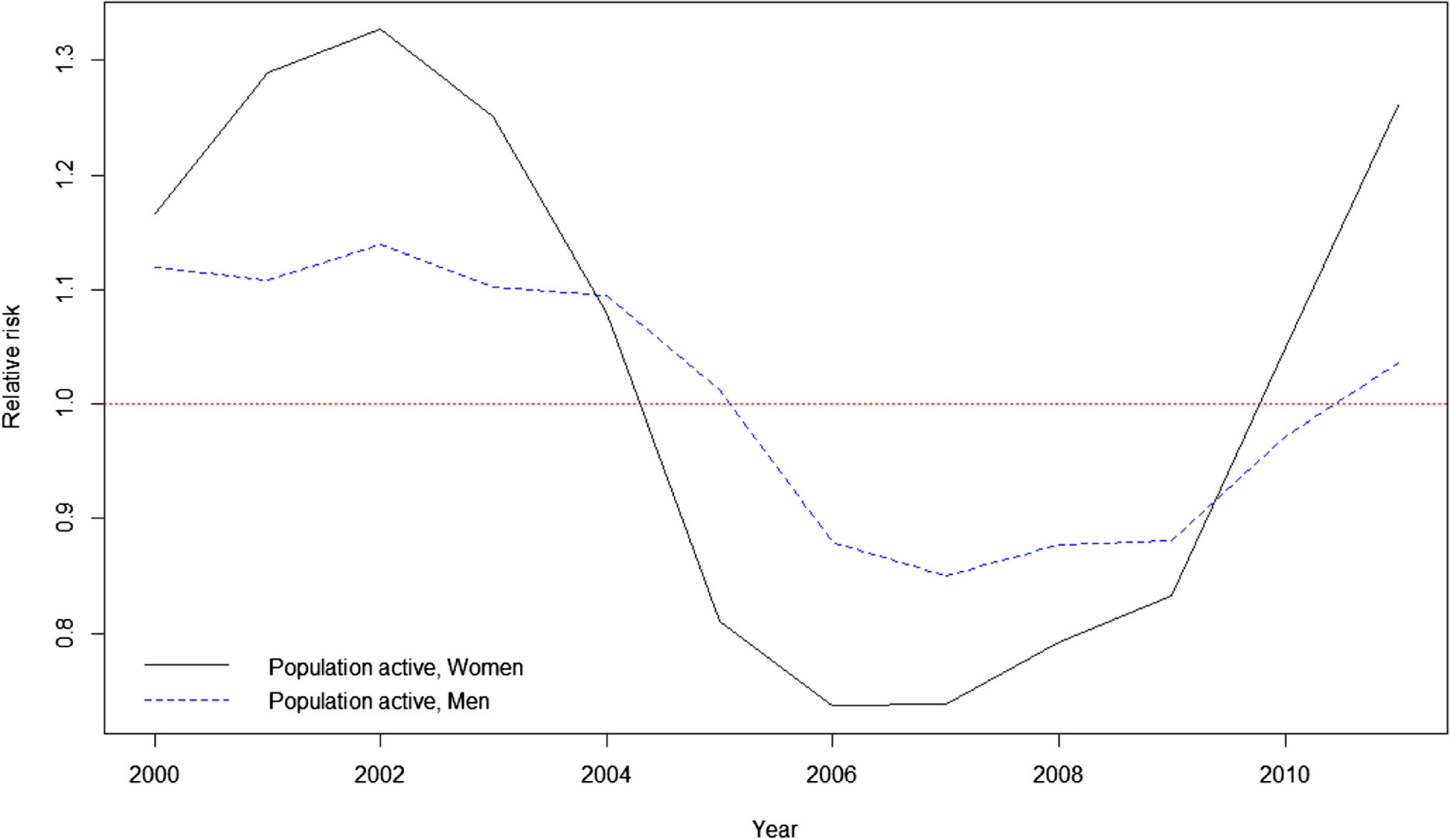
Relative risk of suicides. Population in active age (16–64 years). Dotted lines for men and continuous lines for women

Figure 3 graphically shows the relative risks obtained. For municipalities with more than 10,000 inhabitants (Fig. 3a), relative risks showed a decreasing trend with a sharp drop for females from 2004 to 2006, and then increasing slightly until 2009. From 2009 onwards the increase in risk was higher. The behaviour in males was similar but with some minor differences. As with women, risk among men decreased until 2007 and then increased from 2009 onwards, but more steadily than in the case of women. Figure 3b shows a decreasing trend in risk for both groups from 2006 in municipalities with less than 10,000 inhabitants. While there was a steady drop until 2006 for women, in the case of men there was a slight increase in risk up to 2006 before it then started to decline. In no case were results statistically significant. In Fig. 4, in which the evolution of the relative risk is shown only in the case of the population between 16 and 64, we can clearly see the largest increase in the relative risk for women since 2009.

The results obtained in the estimation of the second model, in which we investigated the possible relationship between unemployment and suicide, are shown in Table 2. The relative risks were not statistically significant in any case.

**Table 2:** Estimation results from the models assessing the effects of unemployment on suicide death rates. Relative risks (95 % credibility interval)

	Municipalities with 10,000 inhabitants or more		Municipalities with less than 10,000 inhabitants	
All	0.826	(0.261, 2.821)	1.005	(0.619, 1.647)
Men	1.823	(0.149, 25.123)	0.908	(0.540, 1.554)
16-64	0.668	(0.045, 10.509)	0.741	(0.436, 1.285)
Women	1.095	(0.012, 203.63)	0.660	(0.323, 1.369)
16-64	0.062	(0.000, 20.338)	0.596	(0.278, 1.307)

## Discussion

In summary, we found that there was an increase, which was much greater in women, in the relative risks of suicide from 2009 onwards. This increase was only found in municipalities with 10,000 or more inhabitants and was only statistically significant for working-age women (16–65 years), although marginally (at 90 % of confidence). In municipalities with less than 10,000 inhabitants, however, relative risks showed a decreasing trend, even from 2009. Note that, because the number of suicides was grouped together, in this case rates were higher than in the case of municipalities with 10,000 inhabitants or more. In no case did we find the unemployment rate to be associated (statistically significant) with the suicide rate. This fact may bring into question a cause-effect relationship between the increase in suicides, from 2009, and the economic crisis, which in the case of Spain began precisely in 2009. In fact, as we mentioned earlier on, the changing trend in suicide rates actually occurs two to three years before 2009.

There is little research that does not find a (statistically significant) association between the total rate of suicide and the economic crisis, as in our case [9, 10, 16, 26, 34]. As previously mentioned, Ayuso-Mateos *et al*. [26] noted that in Spain and Portugal there was no increase, as indicated by other studies [8, 11, 23], but instead a slight decrease in the number of suicides during the economic crisis. In fact, in three of the four studies referenced in this paper that focused on Spain [9, 16, 34], there is no significant increase in the number of suicides during the period of economic crisis (at least until 2011). Karanikolos *et al.* [38] replied to Ayuso-Mateos *et al.* [26], attributing the fact that no increase was found to the total suicide rates, except inthe rates corresponding to men of working age who were most affected by the financial crisis. Furthermore, they point out that the suicides among Spanish people of working age increased by almost 10 % above the underlying trend [25] (the fourth study focused specifically on Spain). Three important caveats should be noted in this case. First, López-Bernal *et al*. [25] estimated a 10 % increase in suicide rates (above the underlying trend) for males (15 years and older) and including males 65 years and older. The increase was 10.4 % for people (males and females, not only males) 15–39 year old and 8.6 % for 40–64 year olds (9.38 % for 15–64 year olds^4^). Second, for the age groups 15–39 years and 40–64 years the estimated increases were not statistically significant (p > 0.1) (the estimated increase for males 15 years and older was statistically significant, p < 0.05). Third, if instead of considering the second quarter of 2008 as the beginning of the crisis in Spain and the standard definition of recession (a decline in the growth rate of GDP over two successive quarters) had been used, the results would have been very different (an even decrease in the overall rate, as evident from Fig. 1 in López-Bernal *et al*. [25]).

In our case, working-aged women (16–64 years), rather than men of that age group, experienced a statistically significant increase in relative risk of suicide during the economic crisis. Our findings are partly consistent with the results in Alameda-Palacios *et al*. [34] for Andalusia (Spain). Although they did not find significant differences between the change in annual rates before and during the economic crisis, 15 to 44 year old males did have an increased rate (equal to 1.21 % per annum), while in women the rate increased both in the 15 to 44 year (equal to 0.93 %) range as well as 45 to 64 years (equal to 0.47 %). These regional differences were also found by Saurina *et al*. [24]. They indicate that there was no statistically significant increase in the number of suicides between 2008 and 2010 in England as a whole, but there were statistically significant increases and decreases in some regions. Catalonia, like Andalusia, is an autonomous Spanish community with its own specific characteristics in terms of customs, the character of its inhabitants, family relationships and its economic situation, meaning that any specific results would differ from those found for Andalusia or for Spain as a whole.

The decreasing trend in suicide relative risks in municipalities with less than 10,000 inhabitants could be attributed to the predominant rural feature of these municipalities. Saunderson *et al*. [39] in their study, although from a few years back, found that in England and Wales female suicides (standardised mortality rates) were highest in urban areas, whereas male suicides presented an excess in rural districts. They pointed out three factors that could explain such differences, namely the difference in suicide methods, the likelihood of communicating suicidal intent and, perhaps, variations in access to psychiatric services [39]. Much more recently, Qin [40] pointed out that poverty, one possible cause of suicide, is an urban phenomenon. According to Qin, living in a more urbanized area reduces the risk of suicide significantly among men, whereas it increases the suicide risk among women [40]. However, neither Saunderson *et al*. [39] nor Qin [40] explain why this phenomenon occurs mostly among women. In fact, before the crisis the Commission of the European Communities warned that urban areas were the scene of multiple forms of discrimination [41], regardless of gender issues. Severe poverty is more prevalent in urban settings as the urban poor are poorer than the poor living in rural areas. Urban poverty is more prevalent among young adults with higher rates of school failure and thus, reduced access to the labour market. Such poverty leads to marginality, especially when coupled with increased drug and alcohol use [42]. In our case, while we have also found that suicide is an urban phenomenon and rising among women, we are reluctant to attempt to provide an explanation for this phenomenon. What is clear, however, is that further research into not only this rise in the suicide rate among women, but also the causes of it, is required and would have implications on health equity.

Moreover, there are many studies showing that poverty does not affect both sexes equally. Women face a higher risk of poverty and generally have greater difficulties in overcoming the situation [43]. The feminization of poverty is usually explained by the difficult reconciliation of work and family life, by the increased presence of women heading single-parent households and the lack of social protection, among other causes [44, 45].

This paper could present some limitations. First, although we work with official data and therefore it is validated data, there could be an under-registration in the number of suicides. If this were the case, we would have underestimated the effect of the economic crisis on the evolution of suicide rates. Second, we work with aggregated data and, apart from the known ecological fallacy; we have not been able to control all possible confounding. Third, the recession in Catalonia (in fact throughout Spain) continued into 2014 and the effects of the crisis continue to be felt today. However, suicide mortality data are not available beyond 2011. The unavailability of such data from 2012–2014, therefore, could be a major constraint. Nevertheless, in Catalonia the crisis actually worsened from April 2010 (coinciding with the introduction of restrictive policies by the Spanish government). In this sense, in this article we have been able to capture this fact. Again, for reasons of data availability, the denominator used for the computation of unemployment rates did not coincide with the active population. However, the active population practically coincides with the population between 16 and 65 years old. Finally, it is not easy to associate urbanity or rurality to municipalities when guided only by size.

## Conclusions

Our first objective in this paper was to ascertain if the increase in the suicide rates in Catalonia, especially since 2010, was statistically significant. While we have shown that the increase in the suicide rate from 2010 in Catalonia was not statistically significant as a whole, it was statistically significant for working-age women (16–64 years) living in municipalities with 10.000 or more inhabitants.

The second objective was to determine if this increase is associated or not with rising unemployment as a result of the economic crisis. Our work rejects this association in all cases and the study suggests that future research into the effects of economic recessions on suicide mortality should take into account inequalities by age, sex and size of municipalities.

As a final conclusion, we believe we have provided evidence that the increase in suicides since the onset of the Great Recession cannot be entirely attributed to it. Undoubtedly, there is a link between poverty and mental health, which in extreme cases may end in suicide. But why it is more prevalent among women and in urban areas are aspects that should not only be considered in health policy, but certainly deserve further research work as well.

## Footnotes

1 The Spanish economy went into recession in the first quarter of 2009 after the GDP fell for two consecutive quarters. Although the Spanish economy emerged from this first recession in the first quarter of 2010, when GDP showed positive growth rates, it slipped back into recession in the last quarter of 2011 (double dip) and did not move out of recession until the third quarter of 2013.

2 A *comarca* can be considered equivalent to a county.

3 If there were more than one municipality with 10,000 inhabitants or more, we subtracted the number of suicides of all these municipalities.

4 Using a weighted average calculated using Tables 1 and 2 in Lopez-Bernal *et al.* [25].
